# Integrating a sense of coherence into the neonatal environment

**DOI:** 10.1186/1471-2431-13-84

**Published:** 2013-05-22

**Authors:** Gill Thomson, Victoria Hall Moran, Anna Axelin, Fiona Dykes, Renée Flacking

**Affiliations:** 1 Maternal and Infant Nutrition and Nurture (MAINN), University of Central Lancashire, Preston PR1 2HE, UK; 2 Department of Nursing Science, University of Turku, Turku, FI 20014, Finland; 3 School of Health and Social Studies, Dalarna University, Falun 791 88, Sweden

**Keywords:** Neonatal, NICU, Family centred care, Sense of coherence, Antonovsky, Service delivery, Parents, Staff

## Abstract

**Background:**

Family centred care (FCC) is currently a valued philosophy within neonatal care; an approach that places the parents at the heart of all decision-making and engagement in the care of their infant. However, to date, there is a lack of clarity regarding the definition of FCC and limited evidence of FCCs effectiveness in relation to parental, infant or staff outcomes.

**Discussion:**

In this paper we present a new perspective to neonatal care based on Aaron Antonovksy’s *Sense of Coherence* (SOC) theory of well-being and positive health. Whilst the SOC was originally conceptualised as a psychological-based construct, the SOCs three underpinning concepts of *comprehensibility, manageability and meaningfulness* provide a theoretical lens through which to consider and reflect upon meaningful care provision in this particular care environment. By drawing on available FCC research, we consider how the SOC concepts considered from both a parental and professional perspective need to be addressed. The debate offered in this paper is not presented to reduce the importance or significance of FCC within neonatal care, but, rather, how consideration of the SOC offers the basis through which meaningful and effective FCC may be delivered. Practice based implications contextualised within the SOC constructs are also detailed.

**Summary:**

Consideration of the SOC constructs from both a parental and professional perspective need to be addressed in FCC provision. Service delivery and care practices need to be comprehensible, meaningful and manageable in order to achieve and promote positive well-being and health for all concerned.

## Background

### Family centered care

Family Centred Care (FCC) is a philosophy, comprising a set of values, attitudes, policies and approaches to services for infants and children with special needs and their families. FCC is an approach that recognises that when an infant is hospitalised, it affects the whole family unit. In addition, as the family are ‘natural advocates’ for their infants [1] the FCC approach places them at the centre of the caregiving processes and procedures [2,3]. Collaboration between professionals and family members to manage infants’ care is at the crux of FCC provision [4]. FCC offers a framework for professionals to care for infants whilst supporting and promoting the emotional and psychological development that occurs within the context of the family. Whilst various concept analyses have been undertaken on FCC, Malusky [5] detailed five defining characteristics: a ‘respectful coalition’ between professional and family members engaging with the decision making process; ‘open communication’ in seeking out perceptions and concerns of families; ‘focusing on family’s strengths’; ‘accepting individuality and diversity’ and recognising families as ‘experts’ in the care of their infant [5], p.27.

Despite emerging evidence about the positive effects of FCC interventions, a recent Cochrane review (updated from review in 2007) by Shields, Zhou et al. [6] of FCC for children in hospital identified only limited benefits of FCC interventions in terms of improvements in children’s clinical care, parental satisfaction and costs within moderate-quality literature. Whilst a more recent systematic review of FCC for children with special health care needs found positive associations of FCC in terms of use of services, health status, satisfaction, access to care, communication, systems of care, family functioning, and family impact/cost [7]; this review only considered interventions that specifically focused on the family-provider partnership as the activity that constitutes FCC; rather than FCC as a model of care.

Nordic countries are considered to have FCC well implemented due to their wider family-centered ethos, high resourcing and specialist staff training [8]. However, research has identified wide differences in the provision of neonatal intensive care units (NICUs) facilities to enable parental involvement [9], which includes ‘space’ for parents to be located near to their infant [10] and parental presence during ward rounds [11]. Other barriers to effective FCC integration relate to socio-cultural beliefs, staffing issues, professional training and attitudes [3]. Shields and colleagues have also identified numerous and repeated problems with the implementation of FCC in various cultures and contexts [12], e.g. parent-staff negotiation of infant’s care. Whilst studies have emphasized the importance of caregiving, information exchange, and relationships within the context of FCC [13], it is argued that FCC is an ideal to be aspired to, however in reality, due to its complex and relational nature and the heterogeneity of context, interventions and populations, it may never be fully practiced [14].

In the following section, Aaron Antonovsky’s *Sense of Coherence* theory is outlined.

### Sense of coherence

Aaron Antonovsky, a medical sociologist, through his work with adults (such as Holocaust survivors) identified how individuals remained physically and mentally healthy despite enduring conditions of severe stress [15]. Antonovsky went on to investigate this phenomenon and proposed a *salutogenic* orientation to health care; an orientation concerned with how people stay healthy and well. His theories stemmed from his fundamental argument against the pathological emphasis in clinical care that dichotomises healthy from sick people; with a pathogenic orientation obtaining a partial or even distorted perspective by only ever considering health in the absence of disease [16]. Antonovsky considered human beings to be ‘terminal cases’ who constantly operate in a dynamic state of disequilibrium due to the omnipotent stress (daily hassles, acute, chronic or endemic stress) that we face. These daily stressors create tension when a need is unfulfilled, a demand has to be met, or a goal has to be reached. Antonovsky advocated that rather than ‘health’ and ‘sick’ operating as pole positions, that a continuum of ‘health ease/dis-ease’ [16] was a more appropriate and meaningful perspective to consider. He believed that whilst a pathogenic perspective was important to determine causality, predictions and interventions; a *salutogenic* approach offered opportunities to explore how individuals are situated on this continuum. His focus therefore turned towards how coping resources enabled people to remain in positive health despite the adversities faced: “Thinking salutogenically not only opens the way for, but compels us to devote our energies to, the formulation and advance of a theory of coping” [16], p.13.

This orientation led Antonovsky to formulate the *Sense of Coherence* (SOC) theory in which he proposed that our SOC is made up of three core components, *comprehensibility, manageability* and *meaningfulness*. *Comprehensibility* is the extent to which our internal and external environments make ‘cognitive sense’. *Manageability* is the extent to which we believe that the resources (internal or external such as finances, social support, environmental factors) at our disposal are adequate to meet the demands of the stressor. *Meaningfulness* is described as the motivational element of the SOC, and relates to whether we perceive the challenge to be worthy of emotional commitment and investment. The logic follows that an individual who perceives the world as meaningful, comprehensible and manageable is more likely to resolve tension compared to someone who views the world as burdensome, unpredictable and overwhelming [15]. Furthermore, while Antonovsky’s theories are cross-culturally applicable, he did consider that an individual’s SOC is constructed within their social, cultural and historical background.

SOC theory has been operationalized as a quantitative tool and subsequently utilised to assess an individual’s coping strategies to life events [16]. In this paper we argue how the three underpinning SOC concepts of comprehensibility, manageability and meaningfulness offer an important theoretical lens to explore how staff and parents experience the NICU. Furthermore, due to the lack of evidence for the effectiveness of FCC, we consider how this approach could enable more meaningful and effective FCC provision. Practice based implications contextualised within the SOC constructs are also discussed.

## Discussion

### Integrating a sense of coherence

In the following sections, we discuss each of the three SOC constructs contextualised by available literature.

#### Comprehensibility

Antonovsky considered that in order to cope with life challenges, we need to understand what the stressors are [16]; thereby emphasizing the need for ordered, consistent, structured and clear information. Difficulties or threats to comprehensibility occur when information is inconsistent, unclear or misleading. Antonovsky refers to these occasions as the ‘noise’ [15] of incomprehensible and unpredictable stimuli, which can destabilise our cognitive interpretations.

Communication is one of FCCs key tenets such as parents being provided with verbal and written information provided at key points (e.g. antenatally, postnatally and discharge) and engagement in decision-making regarding their infant’s progress [17]. On-going communication and information are identified as key features of caregiving requirements [18] and considered important in terms of: reducing parental stress and anxiety; increasing parental self-confidence and sense of control; improving relationships between parents and their infants and facilitating active participation in staff-parent discussions [19-21]. However, studies have identified how a number of parents do not receive sufficient or appropriate information, e.g. Redshaw & Hamilton [22]. In line with Antonovsky’s theories, the ‘noise’ of poor and fragmented information is believed to inhibit open and mutual negotiations and create insecurity, frustration and isolation between parents and professionals [23]. A study by Wereszczak, Miles, et al. [24] revealed how parents of infants cared for in a NICU could recall incidents of receiving incomprehensible information and their questions not being answered for up to three years post-discharge. Furthermore, whilst communication with parents has been identified as a key component in negotiating roles for the care of their infant, studies show that parents may not know what is expected of them [25].

From a professional work-based perspective, Antonovsky considered the need for ‘consistency’; the extent to which our work situation enables us to understand our position within it and what the expectations are of our involvement [16]. Research in this area has identified that a FCC vision becomes comprehensible only when “it is incorporated into the culture and daily practices of the NICU” [3], p.450. One of the most crucial aspects of a change in culture towards a FCC ethos is how staff position themselves in relation to the parents, and their expectations of parental involvement [4,26]. Changes in staff attitudes and behaviours in considering parents as primary caregivers entails a shift of power and role negotiation, which needs active advocacy [3]. Discrepancies between knowledge and practice have also been indicated; with less experienced [27] and better educated [28] staff identified as having a greater awareness, appreciation and application of FCC principles.

#### Manageability

Manageability relates to the extent to which one has the resources “at one’s disposal” to meet the demands of the stressor [16], p.17. Antonovsky proposed the concept of “generalised resistance resources” (GRRs) described as: “any characteristic of the person, group, or environment that can facilitate effective tension management” [15], p.99. Manageability specifically concerns the GRRs (physiological, psychological, contextual and socio-cultural) that are directly under a person’s control or by resources controlled by legitimate others, such as personal networks and professionals. Furthermore, Antonovsky considered that GRRs are dependent on the social class, culture and socio-historical contexts of people’s lives. These GRRs are considered to create prototypical patterns that are crucial to our ability to manage tension, and which subsequently determine our position on the SOC continuum.

According to Antonovsky, experiences of appropriate load balance (underload – overload) are decisive in determining the sense of manageability. Too much pressure, or the feeling that you do not have the resources (e.g. knowledge, skills, materials and equipment) to adequately address the stressor can lead to negative coping (overload); however if a person is not involved in shaping outcomes because his/her resources (or ‘potential’) are ignored or negated (underload), this may also lead to dysfunctional responses [16]. One of the key elements, emphasized by Antonovsky, in relation to a balance between underload and overload, is active participation in decision-making. The literature suggests that the imbalance between overload and underload and negation of parental involvement in decision-making may be a key barrier to FCC implementation.

Underload is repeatedly reported in relation to parental involvement in the NICU in terms of parents’ feelings of being reduced to visitors, not involved in their infant’s care or experiencing a lack of support [29,30]. Studies report how parents use different strategies to cope with underload and reduce the risk of confrontation or being judged by staff by relinquishing care to staff, mimicking staff in order to conform, or alienating themselves from their infant [30,31]. For socio-economically disadvantaged parents this might be even more pronounced. For example, Flacking et al. [25] identified how mothers from low socio-economic backgrounds were more likely to cope with negative interactions with staff through withdrawal or submissiveness.

Overload is illuminated in research that highlights how some parents have to stay with their infants not out of choice, but because of a lack of or less knowledgeable staff [32]. Although parental presence during difficult procedures has been identified as important in building parental confidence [33], parents also express concerns about being involved in procedures that hurt or distress their infant [34]. Furthermore, when parents feel as if they do not have the skills to provide effective care and support or that staff “do it better”, or because of feeling overwhelmed by the task requirements, this can lead to feelings of stress, neglect and being over-burdened [20,25,30,34]. Whilst involving parents as co-partners in care can improve parental confidence and self-esteem [35,36], Antonovsky argued that when others decide everything for us, set the tasks and formulate the outcomes, we become “reduced to being objects” [16], p.92.

From a SOC perspective, high manageability is strongly contingent on high comprehensibility. Therefore, in order to determine whether we have the necessary resources to meet the demands of the stressor, we need to understand what the demands are [16]. A staff survey undertaken by Petersen et al. [27] identified how a lack of organisational guidance and recognition of professional contribution and confidence for autonomous skills debilitated FCC implementation. Other research has indicated how inadequate staffing, multiple caregivers, busy units and limited time may impact upon staff’s capacity to communicate and share information with parents [32]. Shields and colleagues in Australia [37], Sweden [38] and UK [39] also found incongruence between levels of support needs between staff and parents, with staff considering that parents required more support than they did.

Other issues that impact upon manageability of FCC concern the unit layout and organisation, space, comfort and privacy. Whilst almost all NICUs in the UK have rooms in which parents can stay, many have inadequate facilities [22]. Open rooms are common in many NICUs to accommodate technical equipment and facilitate surveillance; however these environments can be stressful for parents [40]; with a lack of suitable facilities contributing to parents’ feelings of unimportance [25], thereby creating ‘underload’. Parental satisfaction with care in a single-family room NICU is higher than in the traditional open-bay NICU [41]. Furthermore, the provision of suitable facilities to enable a parent to stay overnight or even to sit comfortably and in close contact with their babies can enhance breastfeeding success [42]; reduce length of hospital stays [43] and rehospitalisation rates [44]; facilitate bonding and promote access to clinical staff [14] and reduce parental and infant stress levels [45]. Communal areas to obtain peer support and alleviate loneliness through the sharing of experiences are also identified as beneficial [25]. These insights indicate how suitable environmental conditions can enhance FCC provision [41]; which in turn will have a positive impact on parents’ comprehensibility and manageability.

From a SOC perspective, formal social and contextual structures need to be in place such as appropriate involvement, access, environment and equipment in order for an underload and overload balance to be maintained [16]. Whilst progress has been made in NICUs to enable parents unlimited visiting hours, there are still issues in terms of units closing during shift changes, new admissions, and parents asked to leave during medical procedures or ward rounds [46]. In addition, studies have identified that whilst an environment that supports the presence and involvement of parents increases FCC, such as through a 24 hour visiting policy [47], staff concerns about parents visiting at their convenience and interrupted work flow need to be addressed [3].

#### Meaningfulness

Meaningfulness relates to the extent to which life makes sense emotionally. This construct concerns our perception that the demands are worthy of investment and that action is desirable [16]. Whilst Antonovsky perceived that all three SOC components were essential in determining a person’s SOC, meaningfulness was the most important; even if a life-challenge is understandable, and we have resources to meet the challenge, if the challenge is not considered to be worthy of investment, a lower SOC would result.

In relation to a neonatal context, meaningfulness relates to the parent’s motivation and desire to develop relationships with their infants and competence within the parenting role. From a staff perspective, this construct relates to staff engagement with FCC to ensure that parenting goals and needs are attained. In addition, it relates to how meaningful FCC provision is to staff, in terms of ensuring the health and wellbeing of infants and parents.

FCC has developed from attachment theories which emphasize and value the importance of early and continued contact between parents and their infant for bonding, and the development of positive attachment relationships. Research has reported that mothers who spend more time in close contact with their infant are more likely to have higher levels of self-esteem and greater responsiveness to their infant’s signals and behaviours [48]. However, a large body of research has identified psychological difficulties faced by parents when their infant is hospitalised. The enforced separation from their infant, the unexpected alteration of the parental role and the concerns for their infant’s wellbeing have been reported to create high levels of guilt, shame, stress and helplessness [25,35,46,48]. Prolonged separation between parents and infants is believed to enhance parental difficulties in caring for their infant with implications for the parent-infant attachment relationship [49]. Depression, social support, stress, anxiety, self-esteem, maternal well-being and perceptions of motherhood have been found to impact upon mother-infant relationships and interactive behaviours, especially with mothers of preterm infants [50-52].

Meaningfulness can be directly associated with the relational aspects of care; with parent-staff relationships directly impacting on the parent-infant relationships. Research has identified how mothers of infants admitted to the NICU require assurance-type support [18] and emotional based care [53]. Sensitive and collaborative staff-parent relationships based on trust and respect can reduce parents’ feelings of helplessness and powerlessness [25,54,55]. Moreover, they can encourage and facilitate contact between parents and their infants and can help to develop parental competence [56-58]. Families who report positive relationships with staff and consider the care to be more family-centred are more satisfied with their overall care [59]. Furthermore, from a staff perspective, research by Fegran and Helseth [56] identified how a closer relationship between parents and staff led to clinical staff feeling more committed to take care of the vulnerable infants. This research thereby supports Antonovsky’s belief that participation in socially valued decision-making is the source of meaningfulness in one’s work [16]. Studies suggests that almost half of all interactions studied in NICUs could be defined as “instrumental” communication (e.g. action- or task-orientated) or not considered as facilitative for parents to build nurturing relationships with their infants [60], p.61. Staff-parent communication therefore needs to be reciprocal and embedded in the creation of trustful bonds [25].

Research has identified how the highly medicalised NICU environment and clinicians’ focus on technology, rather than active collaboration and engagement with parents, has been found to impact upon the development of parent-infant relationships [35]. When professionals set the boundaries for parental participation and parents feel that the infant belongs more to the hospital than to themselves, this can lead to feelings of exclusion, confusion and anxiety [29,35,48]. FCC as a philosophy was developed to be an antithesis to the paternalistic approach of health-care; however ‘gate-keeping’ practices are still evident within practice [21]. Fenwick, Barclay et al.’s [61] grounded theory study identified how women gained access to their infants through the staff, and the ways in which nurses’ exerted authority and expertise to control these relationships. Therefore, parents’ attempts to obtain a more active role may lead to inter-personal conflict between parents and staff. Parents who do not conform to pre-defined perceptions of ‘good’ parents may be labeled as ‘difficult’, leading to communication difficulties between parents and staff and to staff restricting parents’ involvement in the care of their infants [29]. Paliadelis, Cruickshank, et al. [62] identified how nurses expressed difficulties in implementing FCC due to their perceptions of legal responsibilities in providing clinic care and conceptions of parental abilities. Furthermore, a number of studies have highlighted that when staff perceive parents to be competitors in the care of the infant, they may express a sense of being scrutinized, feeling intimidated by parents and fear of losing power and control [29,35,48,63]. Indeed, the centrality of meaningfulness to the SOC concept is that even if we are able to understand the stressor and have the resources at our disposal to deal with the situation; if we do not sufficiently care or are motivated towards the outcomes, the situation becomes incomprehensible and we lose command of our resources [16].

A further point to emphasize is how relationships with parents may lead to negative implications for staff. For example, Fegran and Helseth [56] explored parents’ and nurses’ experiences of the parent-nurse relationship with six mothers, six fathers and six nurses in NICUs in Norway. Their findings emphasized the tension that clinicians may face in balancing between a professional and personal approach. Close relationships could create situations in which staff felt they were over-stepping the professional boundaries, and therefore attempted to restrict their level of contact with the families [56]. This study as well as the studies undertaken by Berg and Wigert [48] revealed that staff often found the interaction with parents more demanding than the provision of clinically based care. Professionals expressed difficulties in engaging with worried parents and instilling trust and hope [48], with implications for compassion fatigue [64]. A lack of emotional support for staff has been identified as one of the main barriers for FCC implementation [27]. This research thereby illuminates how the meaningfulness of relationships with parents can be negated and minimised in practice.

### Implications for practice

In this section, implications for practice, contextualised within the SOC underpinning constructs have been offered.

To facilitate *Comprehensibility* neonatal staff need to provide parents with understandable, unbiased and timely information [65]. This could involve ongoing, repeated, verbal and written information, starting if possible before the birth, at admission, continued in daily care and as part of discharge procedures. Whilst inclusion of parents within ward rounds has been identified to enhance collaboration amongst team members and enhance parental satisfaction [66], regular opportunities to talk to and listen to parents may be more effective in promoting shared decision making and resolving unrealistic expectations [67]. Information, education and support could be delivered via workshops designed for families. Other communication methods that may be beneficial include parental diaries and binders, dedicated phone lines and web-based programmes [68,69].

Neonatal staff need to be able to comprehend and appreciate parents capacity and willingness to receive information, as well as understand the importance of and ways to deliver the FCC approach effectively to include parents as ‘partners’ in care. This requires on-going training and education. Staff should actively listen to parents, to gauge their level of comprehension and to ascertain which messages the parents want to hear. In the model of FCC described in the POPPY study [70], there was a strong parental involvement from generating principals to defining indicators for implementation. Promising interventions include engaging communication methods [71]; targeted communication interventions with care providers [72]; inclusion of an on-site infant development specialist [73] and use of web based resources and maps to assist individual care providers and family advisors to provide comprehensive FCC to infants and families [74,75]. Involving parents as valid research partners will help develop FCC into a model that is relevant and appropriate for parents’ needs.

To facilitate *Manageability,* in the NICU context*,* parents should be enabled to determine and choose their degree of involvement based on the personal GRRs (social/family networks, economic situation, attitudes and beliefs) at their disposal to ensure that their involvement is both comprehensible and manageable. Cultural, social and environmental conditions of care need to be provided such as adequate staffing resources, the amount of physical space within the units (for parents to be in close contact with their infants, family units encouraging and enabling parents to stay on the unit, facilities for siblings (e.g. crèches or minders) and space to enable privacy for staff as well as parents), flexibility of visiting policies and managerial support. The physical layout of the NICU is crucial, with dedicated family rooms providing space for parents to come to terms with their situation and bond with their infants [76]. However, parent as well as staff considerations should be included in the design of such units [77,78].

There is also a need for counselling and psychology services, specialist staff and parent-to-parent support to address parents’ psychosocial needs [40,79,80]. A family support person can help parents to feel less stressed, more informed, confident and prepared for discharge [19]. Previous research has also suggested how it is important to offer numerous support options for parents, e.g. group support, one-to-one support, and telephone support [81].

*Meaningfulness* in the FCC context may be facilitated by attention to parents’ biological and psychological need to be in close contact with their baby. Whilst physical closeness is easily accessible and natural between healthy new-borns and their parents, NICU geography may disrupt the quantity and quality of contact that parents’ and infants’ experience. As staff operate as the intentional or even unintentional gate-keepers between parents and their infants, the active encouragement of early and repeated physical and close contact is essential to encourage attachment relationships to be formed [82]. Breastfeeding, skin-to-skin contact, massage and involvement in the infant’s care should be facilitated from a relationship strengthening approach to enhance physical and emotional outcomes in infants and parents [36,83]. Furthermore, as trust between parents and staff appears to constitute the basis through which parents feel valued, included and significant in their infant’s lives and care; dedicated key workers should be allocated where possible [25].

Neonatal staff need support and opportunities to reflect on parents’ negative reactions and responses [5]. Suitable training and psychosocial support services should be available for staff to help them develop skills and suitable coping mechanisms to effectively deal with the needs of parents and infants in a NICU [56,84]. As research has identified how relationships between staff and parents impacts upon the relationship between parents and infants, the development of interpersonal skills and availability of suitable support are crucial. Ongoing managerial support to ensure that staff are provided with feedback on their performance and roles may also be beneficial. Antonovsky argued that the opportunity to ‘have a voice in what one does, leads one to invest energy in it’ [16], p.111. Therefore, opportunities for peer reflections and managerial input into practices may well develop, promote and encourage the ‘meaningfulness’ element within staff roles. Indeed as meaningfulness is considered to be the most crucial of the SOC concepts, in terms of how it provides the motivational and cognitive basis for managing and preventing stress [16], consideration of these issues needs to be at the centre of FCC.

## Summary

One of the main criticisms of FCC relates to its lack of definition. Whilst policies, guidelines and resources have been developed, there has been less rigorous evidence about FCC as a model of care. In this paper, we have discussed a new perspective to neonatal care based on Aaron Antonovksy’s *Sense of Coherence* (SOC) theory. Consideration of the SOC constructs from both a parental and professional perspective need to be addressed in FCC provision. This, it is argued, is important for service delivery and care practices to be comprehensible, meaningful and manageable in order to achieve and promote positive well-being and health for all concerned.

## Competing interests

None of the authors have any competing interests in undertaking this work.

## Authors’ contributions

GT conceived the ideas for the paper, and GT and RF were involved in the design and drafting of the initial manuscript. VLM and AA made significant contributions to the content of the paper, in terms of identifying and integrating key literature. FD provided a critical review of the paper, and was involved in the formulation of the practice-based implications. All authors read and approved the final manuscript.

## Pre-publication history

The pre-publication history for this paper can be accessed here:

http://www.biomedcentral.com/1471-2431/13/84/prepub
